# Ngari Virus in Goats during Rift Valley Fever Outbreak, Mauritania, 2010

**DOI:** 10.3201/eid2012.140787

**Published:** 2014-12

**Authors:** Martin Eiden, Ariel Vina-Rodriguez, Bezeid O. El Mamy, Katia Isselmou, Ute Ziegler, Dirk Höper, Susanne Jäckel, Anne Balkema-Buschmann, Hermann Unger, Baba Doumbia, Martin H. Groschup

**Affiliations:** Friedrich-Loeffler-Institut, Greifswald-Insel Riems, Germany (M. Eiden, A. Vina-Rodriguez, U. Ziegler, D. Höper, S. Jäckel, A. Balkema-Buschmann, M.H. Groschup);; Centre National de l’Elevage et de Recherches Vétérinaires, Nouakchott, Mauritania (B.O. El Mamy, K. Isselmou, B. Doumbia);; Joint FAO/IAEA Division of Nuclear Techniques in Food and Agriculture, Vienna, Austria (H. Unger);; Ministère du Développement Rural, Nouakchott (B. Doumbia)

**Keywords:** Ngari virus, viruses, goats, livestock, mosquitoes, Rift Valley fever, Mauritania

**To the Editor:** Ngari virus (NRIV) is a single-stranded RNA virus belonging to the family *Bunyaviridae*, genus *Orthobunyavirus*. The genome comprises 3 segments, the small (S), medium (M), and large (L) segments, which encode the nucleocapsid (N) protein, the 2 glycoproteins Gn and Gc, and the RNA-dependent RNA-polymerase, respectively. Sequence analysis showed that NRIV is a reassortant between Bunyamwera virus (BUNV) and Batai virus (BATV), both from the genus *Orthobunyavirus*. S and L segments derived from BUNV, and the M segment derived from BATV (*1*,*2*). NRIV is more virulent than BUNV and BATV and is associated with hemorrhagic fever. NRIV was first isolated from *Aedes simpsoni* mosquitoes in 1979 and from humans in 1993, both in Senegal (*3*). During 1997 and 1998, humans were affected with hemorrhagic fever diseases in Kenya and Somalia that were caused by Rift Valley fever virus (RVFV) and by NRIV (*2*,*4*).

In 2010, during an ongoing RVFV outbreak in Mauritania, we collected 163 serum samples (62 from camels, 8 from cattle, and 93 from small ruminants) (*5*). RVFV RNA was isolated from serum samples as described previously (*5*). Further molecular testing of the samples was conducted by a SYBRGreen–based real-time reverse transcription PCR (RT-PCR) adapted from a conventional RT-PCR and based on generic primers (bun_group_forw 5′-CTGCTAACACCAGCAGTACTTTTGAC-3′ and bun_group_rev 5′-TGGAGGGTAAGACCATCGTCAGGAACTG-3′) that target a 250-nt sequence of the S segment of Bunyamwera serogroup members (*6*). Real-time RT-PCR was performed in a CFX 96 real-time PCR system (Bio-Rad, Hercules, CA, USA) by using 5 μL RNA with a QuantiTect SYBR Green RT-PCR Kit (QIAGEN, Hilden Germany) in a final volume of 25 μL. Cycling conditions included RT at 50°C for 30 min and 95°C for 15 min, followed by amplification with 44 cycles of 95°C for 15 s, 55°C for 25 s, 72°C for 30 s, and 77°C for 5 s. A melting curve analysis was then performed starting with 95°C for 60 s, and a temperature gradient was conducted from 68°C to 94°C in increments of 0.2°C.

Of the 163 serum samples tested, 2 samples from goats resulted in a positive signal with cycle thresholds of 23 (sample 51) and 28 (sample 65), respectively. Both samples showed similar melting peaks at ≈78.2°C and shared the identical partial nucleotide sequence of the S segment. The sequence belongs to the Bunyamwera serogroup, but the short partial sequence was not sufficient for accurate virus determination and identification. For this reason, both serum samples were used to inoculate cell monolayers of Vero E6 cells that were assayed for virus replication. Only sample 51 displayed a cytopathic effect after 72 h and was further analyzed. We isolated the viral RNA from cell culture with TRIzol reagent (Invitrogen, Carlsbad, CA, USA) and used it to prepare a sequencing library according to a recently published protocol (*7*) but using Illumina adaptors (Illumina, San Diego, CA, USA). We sequenced the resulting library using the Illumina MiSeq instrument with v2 chemistry.

We recovered full-length genome sequences of the S, M, and L segments of the virus and deposited them in GenBank (accession nos. KJ716848–716850). Phylogenetic analysis of complete genome sequences indicated that the virus belongs to the Ngari virus group and showed high homology to previous NRIV isolates in all 3 segments (Figure). As for all previous NRIV strains, the new isolate was highly similar to BUNV regarding the S and the L segment (Figure, panels A, C); the M segment was highly similar to BATV (Figure, panel B).

**Figure:**
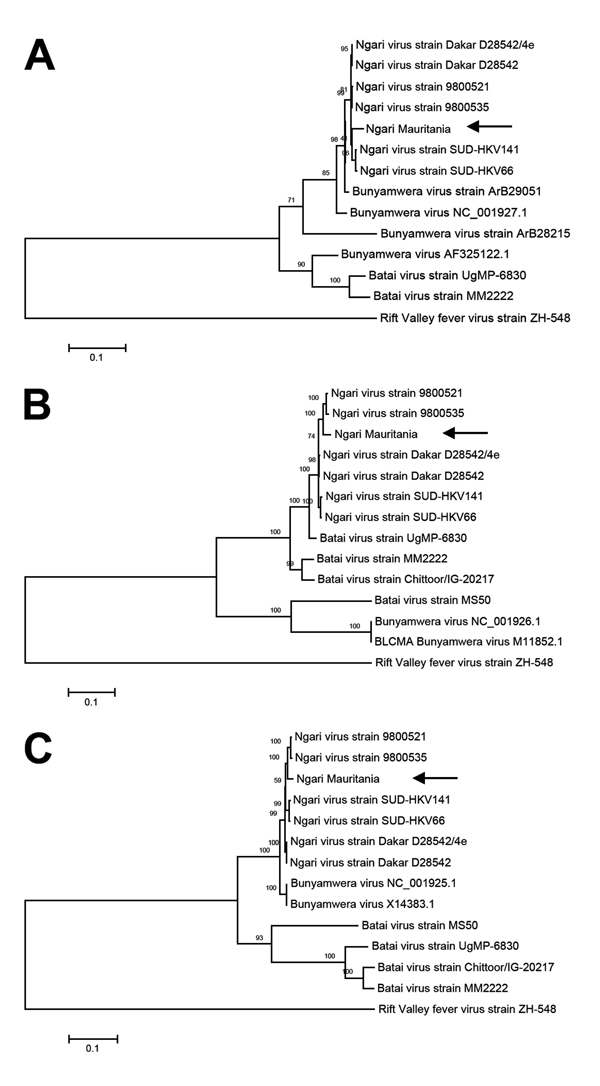
Phylogenetic tree of Ngari virus–derived A) small (975 bp), B) medium (4,507 bp), and C) large (6,887) segment sequences of Bunyamwera and Batai viruses compared with isolate obtained from a goat in Mauritania in 2010 (arrows). The tree was constructed on the basis of the nucleotide sequences of the 3 complete segments by using the neighbor-joining method (1,000 bootstrap replications). The tree was rooted to the sequence of Rift Valley fever virus strain ZH-548. Scale bars indicate substitutions per nucleotide position.

This evidence supports the extension of the range of NRIV infection to goats (complete sequences already had been derived from a human and from mosquitoes [*8*]) and demonstrates the occurrence of NRIV during the 2010 RVFV outbreak in Mauritania. We are aware of only 1 additional report of NRIV-infected sheep (in 1988), also in Mauritania, although no further characterization or isolation has been conducted (*9*). Both NRIV-positive samples were negative for RVFV RNA but positive for RVFV-specific IgG. In addition, sample 51 contained IgM against RVFV (*5*), indicating possible co-infection of RVFV and NRIV. Because both ELISAs rely on detection of antibodies against RVFV N protein, which is highly divergent to the deduced NRIV N sequence, cross-reactivity is highly unlikely but needs to be substantiated. Both samples originated from the Adrar region, which was the center of an unusual RVFV outbreak in Mauritania in 2010 (*10*).

The possible clinical importance to livestock and the circulation of NRIV among mosquitoes, livestock, and humans needs to be clarified. No further information about clinical signs of sampled animals or reports of human NRIV cases is available. Because infection with both RVFV and NRIV induces hemorrhagic fever, affected humans also should be tested for NRIV infection. Further development of specific molecular and serologic diagnostic tools for NRIV should be pursued to obtain more information about NRIV distribution in humans and livestock in Mauritania and other African countries.
